# Feasibility of a randomised controlled trial of remotely delivered problem-solving cognitive behaviour therapy versus usual care for young people with depression and repeat self-harm: lessons learnt (e-DASH)

**DOI:** 10.1186/s12888-018-2005-3

**Published:** 2019-01-24

**Authors:** Kapil Sayal, James Roe, Harriet Ball, Christopher Atha, Catherine Kaylor-Hughes, Boliang Guo, Ellen Townsend, Richard Morriss

**Affiliations:** 1 Division of Psychiatry and Applied Psychology, School of Medicine, University of Nottingham, Nottingham, UK; 2 Centre for Mood Disorders, Institute of Mental Health, Nottingham, UK; 3 School of Psychology, University of Nottingham, Nottingham, UK; 4 Division of Psychiatry & Applied Psychology, School of Medicine, Queen’s Medical Centre, University of Nottingham, Nottingham, NG7 2UH UK

**Keywords:** Depression, Self-harm, Problem-solving therapy, Cognitive behaviour therapy, RCT

## Abstract

**Background:**

Self-harm and depression are strong risk factors for repeat self-harm and suicide. We aimed to investigate the feasibility of a randomised controlled trial (RCT) of remotely delivered problem-solving cognitive behaviour therapy (PSCBT) plus treatment as usual (TAU) versus TAU in young people with repeat self-harm and depression.

**Methods:**

Single-blind multi-centre RCT with an internal pilot, pre-set stop-go criteria and qualitative semi-structured interviews. Eligible participants (aged 16–30 years) were recruited from 9 adult or child and adolescent self-harm and crisis services; had ≥ 2 lifetime self-harm episodes, one in the preceding 96 h; and Beck Depression Inventory-II (BDI-II) score ≥ 17. Participants were randomised (1:1) to either TAU or TAU and 10–12 sessions of PSCBT delivered by mobile phone or video-calling.

**Results:**

Twenty-two participants were recruited (11 in each arm), 10 (46%) completed follow-up at 6 months, 9 (82%) started the PSCBT and 4 (36%) completed it. The study did not meet three of its four stop-go criteria, reflecting considerable barriers to recruitment and retention. Participants had severe depression symptoms: with mean BDI-II 38.9 in the PSCBT and 37.2 in TAU groups, respectively. Three (14%) unblindings occurred for immediate safety concerns. Barriers to recruitment and retention included lack of agency for participants, severity of depression, recency of crisis with burden for participants and clinicians who diagnosed depression according to pervasiveness.

**Conclusions:**

RCTs of PSCBT for young people with depression and self-harm are not feasible using recruitment through mental health services that conduct assessments following self-harm presentations. Clinician assessment following self-harm presentation mainly identifies those with severe rather than mild-moderate depression.

**Trial registration:**

ClinicalTrials.gov (NCT02377011); Date of registration: March 3rd 2015. Retrospectively registered: within 21 days of recruitment of the first participant.

**Electronic supplementary material:**

The online version of this article (10.1186/s12888-018-2005-3) contains supplementary material, which is available to authorized users.

## Introduction

There are strong associations between self-harm and depressed mood and both are significant risk factors for suicide [1–4]. Of those who die from suicide, at least half have a previous history of self-harm and 15% have presented to hospital with self-harm within the preceding 12 months [5, 6]. Based on an update of a systematic review [7] to include 37 randomised controlled trials (RCTs), the National Institute for Health and Clinical Excellence (NICE) guidelines on longer-term management for self-harm made a clinical recommendation that 3 to 12 sessions of psychological treatment involving problem-solving therapy (PST) should be offered to people who self-harm [1]. A Cochrane review of 55 RCTs involving 17,699 participants confirmed that PST and cognitive behaviour therapy (CBT) were effective in reducing the proportion experiencing repeat self-harm, suicidal ideation, depression symptoms and hopelessness over 12 months [8]. However, the quality of this evidence was graded moderate to low [8]. Since PST and CBT are also effective for depression [9], an intervention focussed on people with at least one previous self-harm episode and mild-moderate depression symptoms could be considered to be a clinical priority, given limited resources to offer such treatment to everyone who is assessed for self-harm. The proportion of people engaging with and receiving psychological interventions for self-harm is relatively low at 40–50% [10, 11]. However, mobile phones represent a highly acceptable means for engaging young people in psychological interventions such as CBT for depression [12, 13] and may mitigate clinical factors for non-attendance at face to face appointments that are inherent to depression such as low motivation and poor planning. For people with depression and anxiety, the use of telephone, video-calling or other internet technology to deliver interventions is as effective as face to face treatment, although some techniques require adaptation [14–16]. An RCT of a mobile phone intervention delivering PST, meditation, increased social support and advice on alcohol consumption to people recruited after self-harm reduced suicidal ideation and depression symptoms over 12 months, compared with usual care [17]. Therefore mobile phone and internet delivered PST and CBT might be a feasible way of engaging and delivering such treatment efficiently to adolescents and young adults with depression who self-harm.

The main aim of this study was to determine the acceptability and feasibility of carrying out an RCT of remotely delivered (video-calling or mobile phone) problem-solving cognitive behaviour therapy (PSCBT) plus treatment as usual (TAU) versus TAU in adolescents and young adults with depression who self-harm. Before the study started, as recommended in the Cochrane systematic review of interventions for self-harm for children and adolescents [18], we involved a group of five young people with lived experience (service user group) in the design of the study and the intervention. We also aimed to identify the barriers and drivers to conducting the study and the remotely delivered PSCBT intervention. If key barriers could be successfully identified and addressed, we planned for the internal pilot study to continue into an effectiveness RCT of remotely delivered PSCBT plus TAU versus TAU.

## Methods

### Study design and participants

The e-DASH (electronic - Depression and Self-Harm) study was a parallel group single-blind multi-centre RCT, exploring feasibility and acceptability, using quantitative and qualitative methodologies (Additional file 1: Figure S1). There were two trial arms:

1. Group 1 received treatment as usual (TAU). Participants did not receive any additional treatment to what was routinely offered.
2. Group 2 received TAU and 10–12 sessions of problem-solving using cognitive behaviour therapy (PSCBT) provided by a trained therapist. The PSCBT was delivered by mobile phone or video-calling, depending on the participant’s preference.

#### Inclusion criteria

- Aged 16–30 years
- At least two lifetime self-harm episodes, of which one was in the preceding 96 h. Self-harm was defined as any act of self-poisoning or self-injury carried out by an individual, irrespective of motivation [1]. (On 10th March 2016, the requirement for the self-harm act to be in the last 96 h was removed to improve recruitment (Additional file 2)).
- A score of 17 or more, indicating at least the higher end of mild depression symptoms, on the Beck Depression Inventory version 2 (BDI-II) [19] so that the participant definitely had symptoms of depression.
- Ability to take part in psychological therapy in English.
- Ability to give informed consent.

#### Exclusion criteria

- Clinical judgement of high level of suicide risk, other risk to self or others requiring urgent approaches e.g. psychiatric admission.
- Severe mental disorder (e.g. psychosis, bipolar disorder, substance use disorder or organic mental disorder) as the primary mental health problem, as determined by a structured psychiatric interview - the Structured Clinical Interview for DSM-IV Axis 1 Disorders (SCID) [20].
- Currently receiving structured psychological therapy.

### Study setting and recruitment procedure

Participants were recruited from 13th February 2015 to 22nd April 2016, in the East Midlands region of England, through: 1) adult or child and adolescent mental health services that assess people in emergency rooms or hospital wards following a self-harm presentation; 2) adult or child and adolescent community mental health services that see people with depression and self-harm; 3) a third sector organisation providing interventions and support to people who have self-harmed. In keeping with the Clinical Trials recommendation, the trial was registered within 21 days of recruitment of the first participant. Trial registration: ClinicalTrials.gov (NCT02377011).

The procedure of recruitment followed the successful approach described by Brown et al. [21], except we allowed referral within 96 h of the self-harm episode rather than only 48 h. This 96 h referral window reflected the maximum time duration between a self-harm presentation occurring outside normal working hours and the next working day (e.g. following a long weekend). It also helped to minimise the time duration between the self-harm episode and obtaining participant consent to participate in the study and starting the intervention. The initial approach came from the clinician or case worker who had been informed about the study by the researcher and been given a study information pack containing a checklist describing the inclusion and exclusion criteria and case vignettes to help them consider whether an individual was suitable for the study. The information pack also contained a participant information sheet and study leaflet to pass on to the individual. Clinicians or case workers were asked to refer any individuals who met the study inclusion criteria and were in agreement for their details to be passed on to the researcher. Following this, the researcher attempted to contact them and arrange a suitable time to meet, explain the study and obtain informed consent. The consent included contact details of up to three family or close friend contacts that a participant was willing to give to the research team if there were safety concerns (see below). If, during the course of the study, the participant’s level of risk escalated (e.g. loss of contact with frequent or intrusive suicidal ideation at the last contact) the study safety plan was implemented with the study therapist or researcher contacting the three people nominated at the outset. In addition, if deemed necessary, standard National Health Service (NHS) risk management procedures were implemented and the participant’s usual care clinician was contacted. This could include their GP, case worker and/or mental health professional. If the participant met inclusion criteria, the researcher completed other baseline assessment measures.

To compensate participants for their time they were given a shopping voucher following the completion of the baseline assessments. Participants who remained in the study until the end (6 months) received another voucher to thank them for taking part. Similarly, study participants completing the qualitative interview (see below) were offered a voucher as an inconvenience allowance for their participation. Travel expenses were offered for any visits incurred as a result of research participation.

### Intervention

The intervention involved a modified version of a successfully trialled face-to-face PSCBT focussing on internalised problems (e.g. hopelessness) as well as external problems (e.g. relationships) for people who had self-harmed [21, 22]. The intervention is for depression, especially hopelessness in particular, to prevent further self-harm and suicide attempts. The primary outcome measure is a depression rating (see below). In the current study, the delivery model was modified so that the intervention was delivered remotely either by mobile phone or video-calling (WebeX; https://www.webex.com) according to participant preference. Once randomised to the intervention arm of the study, participants (according to their preference expressed at recruitment) received a text message, email or telephone call from the study therapist inviting them to participate in a 1 hour pre-therapy session (Additional file 3). PSCBT was delivered over 10–12 sessions by a cognitive behaviour therapist (CA) experienced in delivering PSCBT for people who self-harm (Additional file 4).

### Outcome measures

The primary outcome measure was the self-rated Beck Depression Inventory-II (BDI-II) [19]; a score of 0–13 indicates minimal depression, 14–19 mild depression, 20–28 moderate depression and 29–63 severe depression.

Secondary outcome measures were the:

- 9 item Personal Health Questionnaire (PHQ-9) measuring depression [23]
- Beck Hopelessness Scale [24]
- Generalised Anxiety Disorder Assessment (the GAD-7) [25]
- Columbia Suicide Severity Rating Scale (CSSRS) to assess suicidality [26]
- Work and Social Adjustment Scale (WSAS) [27]
- EQ-5D 5 L as a measure of health utility [28]

In addition, the 12-item Urgency Perseverance Premeditation Sensation-seeking (UPPS) Impulsive Inventory urgency subscale was used at baseline to measure trait ability to resist impulses to behave irresponsibly in response to negative emotions such as depression, anxiety, or anger [29]. In the presence of depression or anxiety symptoms, this is predictive of self-harm behaviour over 4 weeks [30].

### Randomisation

Consenting participants’ details were entered onto a web-based randomisation system which was password-protected and conducted through a registered Clinical Trials Unit (CTU; Queen’s Medical Centre, Nottingham). This generated an email containing details of group allocation which was sent to the trial co-ordinator or administrator who then sent details of the allocation to the CBT therapist. The researcher, blinded to randomisation, only received an email stating that the participant had been randomised. Participants in the study were randomly allocated to either the intervention group (PSCBT plus TAU) or TAU only. Participants were allocated with equal probability to each treatment arm with stratification by NHS Trust or third sector organisation. The participant was assigned to treatment by a computer-generated pseudo-random code using random permuted blocks of varying sizes of two, four or six, created by the Nottingham CTU in accordance with their standard operating procedure and held on a secure server. Only the trial co-ordinator, or their nominee, had password access to the randomisation data. All unblindings were recorded.

### Sample size

Based on the Slee et al. study [31], with BDI-II mean scores for PSCBT 31.4 (standard deviation (sd) 12.9) and TAU 34.7 (sd 14.0) at baseline, and for PSCBT 16.6 (sd 13.7) and TAU 28.6 (sd 18.6) at 6 months respectively, 60 participants per arm were required to detect the minimum clinically important difference in BDI-II score of 10 points (28.6 vs 18.6) at 6 months, with 80% power and two-tailed 5% significance level, assuming 30% loss to follow-up [21].

### Assessment of feasibility of the RCT

The following criteria were set before the study started to indicate that both the study and intervention were feasible for a full RCT:

1. Recruitment of 5 participants per month;
2. Collection of data from 70% participants on primary outcome at 6 months;
3. Engagement of 80% participants allocated to the PSCBT intervention;
4. Retention of 60% participants for 10–12 sessions of the PSCBT intervention.

A detailed log was collected on all attempts by the research team (1.8 whole time equivalent (wte) research staff who were blinded to the intervention arm) and 0.4wte CBT therapist to contact participants. Research team members were in regular contact with the recruiting clinicians and services, including physically attending the service sites on a regular basis, to facilitate and support participant recruitment.

An independent scientific committee monitored progress of the project at 14 months, making a recommendation whether to progress to the full RCT, with the final decision made by the Director of the funding body.

### Ethics approval

Ethics approval was obtained from the National Research Ethics Service (NRES) Committee East Midlands - Nottingham 1, UK on 24th September 2014 (REC reference: 14/EM/1084).

### Statistical analysis

Descriptive statistics using Stata 13 for variables are presented by treatment arms at baseline and at follow-up with mean (sd) for normally distributed variables, median (range) for skewed variables and frequency (percentage) for categorical variables.

### Qualitative study

Individual qualitative interviews were carried out with 15 staff members from recruiting services (eight adult services clinicians, four children and adolescent services clinicians and three service user led organisation staff), two study non-participants (people who were invited to take part in the RCT but declined), and five participants from the RCT. Interviews were semi-structured using a topic guide and were digitally recorded and transcribed by an experienced qualitative researcher (JR) who helped to recruit participants but was blinded to the allocation and delivery of treatment until all follow-up was complete. All participants gave written consent to the qualitative interviews. Supplementary interviews and feedback were obtained from a service user group of five young people with a history of self-harm who did not take part in the RCT. They did not know the study findings when they were interviewed about the barriers and drivers to taking part in the study (the protocol was presented to them). The qualitative study was designed to understand barriers and drivers to the recruitment and retention of participants to the study. Field notes were also collected by researchers in relation to barriers and drivers to the recruitment and retention of participants, and by the CBT therapist on barriers to the uptake and delivery of the PSCBT interventions.

Analysis of the qualitative data (by JR) proceeded in parallel with the interviews and was inductive. Interviews were transcribed verbatim and coding was informed by the accumulating data and continuing thematic analysis [32, 33]. A proportion of the data was thematically coded by a second experienced qualitative interviewer and results were discussed by both to draw out an emerging thematic analysis. Findings were also discussed with a panel of multi-disciplinary staff including psychiatrists and nurses.

## Results

### Participant flow and data

**Fig. 1:**
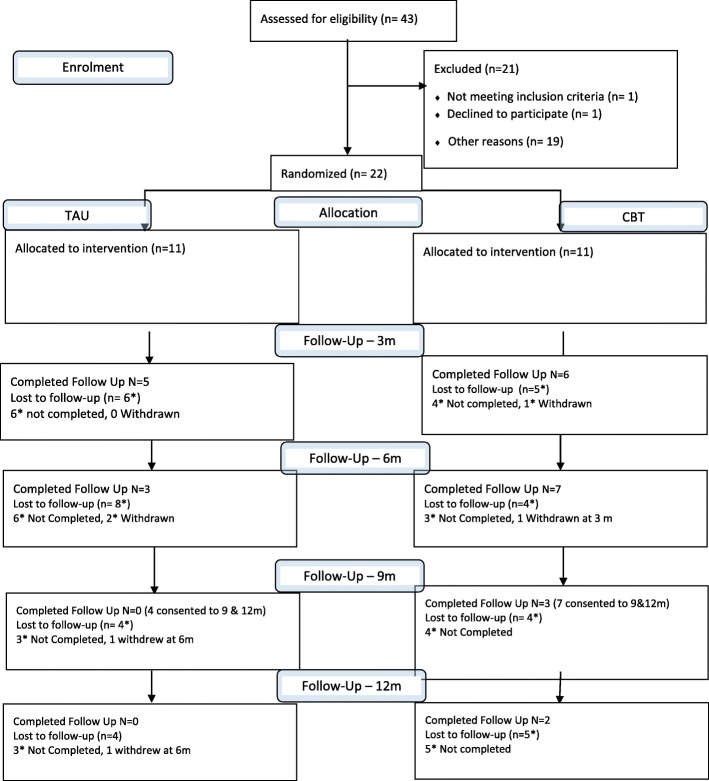
Consort flow diagram

Figure 1 shows the flow of participants through the study. Over 14 months, the study received 43 referrals from 9 services, and recruited 22 participants, three of whom were recruited through a third sector organisation in the last month of recruitment (Table 1). Eleven participants were randomised into each arm. The study fell considerably short of the recruitment target of five participants per month, achieving 3.1 referrals per month with randomisation of 1.6 participants per month. Only 10 out of 22 (45.5%) participants completed follow-up at 6 months so the study did not meet the second pre-set criteria for feasibility, set at 70%.

**Table 1:** Recruitment summary

NHS Trust	Research Site	Age range seen by service (years)	Months Actively Recruiting	Total Referrals	Rejected / Unable to reach	Consented to Study
Trust #1	Adult Service A	17+	14	6	1	5
	Adult Service B	17+	8	13	8	5
	Child & Adolescent Service A	up to 19	7	3	0	3
	Child & Adolescent Service B	16–18	7	0	0	0
Trust #2	Adult Service C	16+	9	11	7	4
	Adult Service D	16+	9	1	0	1
	Child & Adolescent Service C	up to 18	9	3	2	1
	Adult Service E	18+	4	0	0	0
N/A	Third Sector Organisation	Any	< 1	6	3	3
			Total	43	21	22

Additional file 5 highlights the amount of time and effort taken to recruit the 22 participants; there were 181 attempts to contact 43 participants (4.2 attempts per participant) at baseline. Additional file 6 shows the reasons recorded by the research team for non-participation, from field notes on the non-participants. Additional file 2 reveals the barriers identified by the research team during recruitment to the study and steps taken to address these barriers. A study amendment to recruit participants who had not recently self-harmed (i.e. more than 96 h after their last self-harm episode), through a third sector organisation, improved recruitment in the final month.

Table 2 describes the baseline characteristics of the participants. The baseline study assessment was completed a median (range) 13 (0, 46) days after referral. In 12 (55%) participants, the most recent self-harm episode had been a suicide attempt. Participants had severe depression symptoms at baseline with mean (sd) BDI-II 38.9 (13.1) in the PSCBT and 37.2 (11.0) in the TAU groups respectively; 16 of the 22 participants fell into the ‘severe depression’ category, (of whom five had a BDI-II score of 54 or above), five had moderate depression and one mild depression. No referred individuals were excluded from the study. Table 2 shows mean scores (at baseline) reflecting moderately severe hopelessness, anxiety, work and social adjustment difficulties and poor perceived health compared to established general population norms for the United Kingdom. Additional file 7 shows participants’ outcome scores.

**Table 2:** Baseline characteristics of participants in the e-DASH RCT

Baseline characteristic	TAU (*n* = 11)	TAU + PS CBT (*n* = 11)	Maximum
Age, median (range) years	20 (16–28)	21 (16–30)	
Gender, female, n (%)	8 (73)	9 (82)	
Employment: working/student, n (%) Unemployed/not in education, n (%)	6 (54) 5 (45)	9 (81) 2 (18)	
Marital status, single, n (%)	7 (64)	8 (73)	
Ethnicity, white British, n (%)	11 (100)	10 (91)	
BDI-ll, mean (SD)	37.2 (11.0)	38.9 (13.0)	63
PHQ-9, mean (SD)	19.3 (4.9)	19.9 (5.3)	27
GAD-7, mean (SD)	14.7 (3.9)	14.0 (5.6)	21
Beck hopelessness scale, mean (SD)	13.8 (5.8)	13.7 (5.3)	20
UPPS, urgency subscale, mean (SD)	21.7 (5.9)	22.6 (5.0)	48
CSSRS, lifetime ideation, mean (sd)	4.4 (1.3)	3.9 (0.8)	5
CSSRS, lifetime intensity, mean (sd)	18.2 (5.8)	12.2 (5.3)	25
CSSRS, 3 month ideation, mean (sd)	3.3 (2.0)	3.1 (1.2)	5
CSSRS, 3 month intensity, mean (sd)	13.8 (7.8)	10.2 (7.1)	25
CSSRS, suicidal behaviour, lifetime, actual, n (%)	10 (91)	8 (73)	
CSSRS, suicidal behaviour, lifetime, interrupted, n (%)	6 (54)	2 (18)	
CSSRS, suicidal behaviour, lifetime, aborted, n (%)	7 (64)	3 (27)	
CSSRS, suicidal behaviour, lifetime, preparatory, n (%)	7 (64)	5 (45)	
CSSRS, suicidal behaviour, past month, actual, n (%)	7 (64)	5 (45)	
CSSRS, suicidal behaviour, past month, interrupted, n (%)	3 (27)	0	
CSSRS, suicidal behaviour, past month, aborted, n (%)	3 (27)	2 (18)	
CSSRS, suicidal behaviour, past month preparatory, n (%)	4 (36)	2 (18)	
CSSRS, actual, most lethal, mean (sd)	1.3 (0.8)	2.0 (0.5)	5
CSSRS, potential, most lethal, mean (sd)	1.5 (0.7)	2.0 (0)	5
WSAS, mean (sd)	20.1 (10.5)	24.9 (8.9)	40
EQ-5D 5 L, mobility, mean (sd)	0.27 (0.47)	0.36 (0.81)	5
EQ-5D 5 L, self-care, mean (sd)	0.55 (0.93)	0.18 (0.40)	5
EQ-5D 5 L, usual activities, mean (sd)	1.18 (0.75)	1.45 (1.37)	5
EQ-5D 5 L, pain/discomfort, mean (sd)	0.64 (0.92)	1.18 (1.25)	5
EQ-5D 5 L, anxiety/depression, mean (sd)	2.09 (1.04)	2.27 (1.17)	5
EQ-5D 5 L, health today, mean (sd)	51.5 (16.2)	54.0 (25.7)	0

*TAU* Treatment as usual

*PSCBT* Problem-solving using cognitive behaviour therapy

*BDI-II* Beck Depression Inventory version 2

*CSSRS* Columbia Suicide Severity Rating Scale

*GAD-7* Generalized Anxiety Disorder scale 7 item

*PHQ-9* Personal Health Questionnaire 9 item

*UPPS* Urgency Perseverance Premeditation Sensation-seeking

*WSAS* Work and Social Adjustment Scale

There were no adverse incidents in the study but there were unblindings of the research team on safety grounds on three occasions, either at baseline or before the first follow-up assessment (two in the TAU and one in the PSCBT groups): one due to severe self-neglect secondary to severe depression in a participant with no support network; one continuing to exhibit suicidal behaviour with high medical risk; and one admitted to hospital because of escalating self-harm following school exclusion. In each instance, the study team had no ethical alternative but to unblind and signpost to mental health services since the participant had been unable to obtain help from clinical services through their own efforts. For the participant in the PSCBT arm, the unblinding led to withdrawal from the study before the intervention started.

### Participants in the intervention arm

The CBT therapist contacted the 10 participants allocated to 10–12 sessions of PSCBT (as described above, the eleventh was withdrawn from the study) on 374 occasions (37.4 attempts per participant; Additional file 8). Four (36%) participants completed the PSCBT intervention. Five participants dropped out of or discontinued treatment - three at session 3 (one moved out of area, one was told by their mental health professional that PSCBT was inappropriate for them, one unknown reason), one at session 5 (no longer wished to work with male therapist) and one at session 8 (reason not given). One participant did not respond to any contacts and one was withdrawn, as noted above. Hence, nine (82%) participants started PSCBT and the study met feasibility criteria 3 (80% participants receive PSCBT) but not criteria 4 (60% participants complete 10–12 sessions of PSCBT) for progression to the full RCT. Additional file 9 shows the often multiple and over-lapping barriers to engagement and retention in the PSCBT intervention; these included severity of depression and added burden of therapy, restricted finances for phone or internet access, data protection and confidentiality because other family members could access information and problems with buffering during video-calling.

### Decision to stop study recruitment

As planned, the independent Scientific Committee reviewed study progress at 14 months. The Director of the funding body considered their recommendations and stopped recruitment as the study was deemed not feasible. It had not met three of the four pre-set criteria for feasibility and there were also problems with unblindings due to participant safety.

### Qualitative interview data

The following three themes emerged, illustrating barriers to recruitment of participants in the study: Theme 1, identification of depression by clinicians (Table 3); Theme 2, agency and burden on service users at the time of crisis and severe depression (Table 4); Theme 3, burden for clinical staff (Table 5). Themes 1 and 2 were also barriers to retention in the study because of the severity of depression (recognition only of severe or moderately severe depression and the burden of study participation for those with severe depression).

**Table 3:** Barriers to participation and retention in study - Identification of depression by clinicians (Theme 1)

As the vast majority of assessments are conducted by self-harm clinicians at the time of first presentation to accident and emergency services, patients were often difficult to assess because they were distressed and intoxicated from alcohol, illicit drugs or substances ingested in the overdose. *“Most people who we get are very complex so trying to pick out depression when they have substance misuse problem, personality issues, so getting a very clear depressive diagnosis can be tricky.” (NHS Adult Site A)* While clinicians accepted that individuals were often presenting with symptoms of depression, they usually saw these as a response to life events and so should not be used to diagnose clinically significant depression. Depression was reserved for participants who had pervasive depression that was not related to life distress and was therefore uncommon. *“People overly use depression, very loosely, that isn’t depression at all, they might be unhappy about something, they might be distressed about a particular circumstance, but actually when they are not in that circumstance, and when there’s somebody else, it’s not a pervasive mood that lasts in all environments, all of the time” (NHS Child and Adolescent Site C)* Instead clinicians from CAMHS (Child & Adolescent Mental Health Services) often conceptualised emotional dysregulation instead of depression. *“I don’t tend to assess many young people who are depressed, I tend to assess people who are dysregulated, emotionally and low in mood” (NHS Child & Adolescent Site C)* Clinicians in adult services diagnosed underlying trauma or personality disorder instead of depression. *“They may hit the threshold for depression but they might not be getting a kind of diagnosis of clinical depression because it might be more sort of secondary to kind of trauma or abuse or it might be kind of diagnostically-wise personality disorder” (NHS Adult Site E)* For some clinicians, the tendency to conceptualise patients with depression who self-harmed as having personality disorder was accentuated by their reported intentions of harming themselves or others. *“I’ve lost count, it’s almost every other day at least with someone with a certain personality disorder – if you do not do this I will do this, you know I will kill my girlfriend or I will hang myself.” (NHS Adult Site B)*

**Table 4:** Barriers to participation and retention in study - Agency of and burden on service users from the crisis and severe depression (Theme 2)

Clinicians pointed out that the decision to seek help from accident and emergency departments after an episode of self-harm was often made by family members or carers who persuaded the person who had self-harmed to get medical help, not necessarily psychological help. As a result they might have superficially agreed to participate in the research to get home quickly. *“A lot were wanting to put it behind them, people were a bit like, you know, no most people accepted actually, and would sort of say “yes yes yes”, having done it myself – you say “yeah yeah yeah I’ll fill in that thing for the prize draw” and you’re just saying to kind of move on.” (NHS Adult Site B)* Clinicians also reported that they tended to receive the same response if they offer an intervention, regardless of whether it is part of a research study. *“People just genuinely want to forget about it and don’t want to… they might agree to it at the time, but then when they think about it at home afterwards its, no I actually don’t want to be involved in psychiatry” (NHS Adult Site C)* Participants highlighted that both the initial response to participation in the study or psychological treatment reflects their mood. *“Personally, in some of my lowest times, had I been asked by a doctor to take part in something like this, I simply would not have gone back to the doctor. Not because I don’t feel that work like this is important but because when I’m struggling I don’t want to be given more pressures, no matter how slight, and I don’t want to be boxed into another part of the system when it’s taken all my energy just to take this one small step”. (Service User Group)* One participant highlighted that they took part because their depression symptoms were not too severe but would not have engaged if these symptoms were more severe. *“I think it was probably the perfect time, because I wasn’t, I wasn’t like down completely down in the dumps - I wasn’t like the best I’ve ever felt - I was sort of like in the middle, normal sort of*…*if completely down in the dumps, I probably would have took it on but it would have been a case of not answering my phone or you know missing appointments and forgetting and being you know still in bed and you know not hearing the door kind of thing, that’s, that’s what it’s like so.” (Participant, TAU)* Other participants struggled to engage in the study because of the recent distress leading to their self-harm episode while another chose not to participate in the study for this reason. *“I think it was a bit too soon because I was still in, obviously, a lot of shock… I was upset. They could have left me a couple of days and then come to see me.” (Participant, Intervention Arm)* *“I think I was too nervous at the time to talk about it… maybe it was probably just too soon”. (Non-participant)* However, CAMHS clinicians noted that many children and young people will soon want to put their self-harm behind them and could be difficult to engage two to three days after the self-harm episode. *“That window of opportunity I think is quite brief that you can actually talk about it still in an objective way, and I think the longer you leave that – certainly over two or three days it’s gone.” (NHS Child & Adolescent Site A)*

**Table 5:** Barriers to participation and retention in study - Burden for clinicians in self-harm services (Theme 3)

Clinicians in self-harm services reported some problems with recruitment to research that is common to most RCTs carried out in busy clinical services. *“I’ll be honest, forgetting because you are doing so much anyway in an assessment and you have so much to sort out and 9 times out of 10 you’ve got another assessment to go. And the amount of times afterwards I thought argh damn e-DASH – to be honest that happened a massive amount of time, a huge amount of time, it’s the last thing on your mind when you are doing it and I know I’m not the only one – and I was particularly bad for that to be honest” (NHS Adult Site B)* However, mental health assessments with people who are in crisis and have self-harmed can be emotionally charged. Recruitment to an RCT can then seem inappropriate. *“Sometimes you don’t want to risk the fact you had got them to a place that you had got them to a place where they were happy and calm and I felt a reluctance to add some extra stuff on in case it kind of cheapened the interaction.” (NHS Adult Site C)* Randomisation can then add to staff burden, bringing out a wish to protect the patient from additional burden. *“I felt, I used to do it, but I felt like a sales person, I know how I feel when people are trying to sell you something and you feel kind of pressured, I just felt it was the wrong time to be, quite distressed, a lot of the time it’s the middle of the night and I hate seeing people then, at 3am or 5am in the morning, it’s a bit weird doing a mental health assessment when they’ve been up all night, distressed, so then to try and say “oh, we’re doing this research thing where you might get picked but you might not”, trying to sell it in a positive light. Although I used to do it and people signed up for it, it felt it wasn’t the right time to be doing it.” (NHS Adult Site C)*	

## Discussion

### Main findings

Despite considerable efforts and resources and a systematic approach to identifying and addressing barriers to recruitment and retention, recruitment of young people with depression and repeated self-harm from self-harm and wider services was not feasible to an RCT of remotely delivered problem-solving treatment. During the internal pilot study, three out of four pre-set criteria for feasibility were not met; these were low recruitment, low retention in follow-up and low retention in the remotely delivered PSCBT intervention. In addition, three (14%) participants had to be withdrawn for safety reasons. Therefore the study was not continued. Three adult participants were recruited in the last month of recruitment through a third sector organisation indicating that recruitment of young people with depression and self-harm, more than 96 h after the last self-harm episode, might be possible in the community. Qualitative interviews (see Tables 3, 4 and 5) with participating and non-participating young adults also indicated the possibility of such recruitment but staff interviews suggested that such an approach might not be as effective for adolescents.

Mixed quantitative and qualitative methods showed that most barriers to recruitment and retention during both the intervention and study follow-up periods appeared to be intimately connected with the nature and practice of mental health assessment in self-harm services. These barriers included lack of agency of participants. Clinicians reported that many people who attend emergency rooms do so reluctantly at the insistence of family and friends for medical rather than psychological help. Therefore although they initially agreed to participate, they may not have felt committed to this - hence, only half of those who initially agreed to participate were eventually recruited to the study.

Additional barriers to recruitment and retention were the clinical identification of participants with more severe depression, and the emotionally charged atmosphere in which self-harm clinicians carry out assessments. According to previous NICE Guidelines for self-harm [34], people who self-harmed were usually admitted overnight to hospital for next day mental health assessment once the patient was medically fit, no longer intoxicated with alcohol or drugs and less acutely distressed. Pressures on bed use in acute hospitals coupled with rising rates of self-harm presentations mean that such practice now rarely occurs [35]. However, self-harm services are still expected to provide comprehensive risk and biopsychosocial assessments, identifying ongoing mental health and social problems, soon after self-harm presentation to the accident and emergency department [1]. Under these circumstances, clinicians reported that people who self-harm are difficult to assess in terms of their ongoing mental health (including depression) and social problems because they are often still intoxicated by alcohol, drugs or the substance(s) they had taken as an overdose and/or in a heightened state of emotional distress. The high emotional distress experienced around the time of self-harm was highlighted by clinicians as an important reason for not discussing the study with potential participants - clinicians were primarily focussed on defusing emotional distress and did not want the additional burden of explaining a research study to potential participants.

In this context, depression was difficult to diagnose and depression symptoms were usually conceptualised as being secondary to life stress, emotional dysregulation or personality disorder. Depressive disorder was diagnosed rarely by clinicians working in these services; they made the diagnosis of depression on the basis of pervasiveness and lack of clear relationship with life stress. However, pervasiveness of low mood and lack of reactivity of mood are typical of melancholia and other severe forms of depression [36]. Most of those recruited had severe depression according to the BDI-II when assessed by the research team 2 weeks later. They also had moderately severe hopelessness, anxiety and social impairment. However, in contrast to the views of clinicians, they did not report traits of impulsivity in relation to negative emotion, as is more typical of people with borderline personality disorder or emotional dysregulation. The UPPS urgency scores at baseline in this study were below those for a control group and people with borderline personality disorder [37]. Furthermore, it is a matter of concern that several participants deteriorated rapidly with further psychosocial crises to a level that required withdrawal from the study.

The severity of depression at a time of psychosocial crisis was also a major barrier to the retention of participants in the PSCBT intervention and follow-up as was fear of negative reactions of others [38, 39]. Participants in this RCT were more depressed than in previous successfully conducted RCTs of PSCBT in self-harm where retention rates in both treatment and follow-up were considerably higher [21, 31]. In these RCTs, initial assessment of the severity of depression was conducted by the research team rather than clinicians working in self-harm services. However, one problem with such an approach is that front-line clinicians in these services may not be able to apply the results of such research because they do not recognise less severe depressive disorder.

### Methodological issues

Strengths of the study included the use of pre-set criteria for the feasibility and acceptability of the RCT and the intervention to mitigate the possibility of non-completion of the trial, the collection of detailed accounts of efforts made to recruit and retain participants without burdening the services from which participants were recruited, and detailed consideration of barriers and drivers to recruitment during the internal pilot. Despite these, we could not improve referral rates to the study. A further strength was the use of qualitative interviews comparing and contrasting the experiences of clinicians in these services as well as eliciting perspectives of participants and non-participants, supplemented by feedback from a service user group of young people with a history of depression and self-harm (see Tables 3, 4 and 5).

As well as the clear recruitment and retention difficulties, another limitation of the study design is that we do not have information on participants who might have been eligible for the study but were not referred to it. Therefore we have to infer that if depression was conceptualised differently by clinicians, we could have had many more participants who met the study inclusion criteria, based on previous observational studies of the prevalence of depression in people who self-harm [2, 4]. Our data do not indicate how people with milder levels of depression with repeat self-harm could be identified and whether such people have higher levels of impulsivity, as clinicians suggested.

We did not test the intervention in a case series study design before conducting the RCT for two main reasons. First, we worked with, and recruited some participants from, a third sector organisation providing interventions and support to people who have self-harmed. This organisation was already providing remotely delivered CBT to people with a history of self-harm, many of whom also had depression. However, in contrast to this study, the intervention was offered when the participant felt ready to engage rather than shortly after the index self-harm presentation. Second, by carrying out the internal pilot in the way that we did and collecting as much information as we did, we have gained a much better idea of the barriers and facilitators to conducing an RCT than a case series design (without attempts to recruit at scale and understanding the impact of randomisation) would have offered.

In terms of the optimal timing of the clinician approach for participation in the study, there were contrasting views amongst clinicians, study participants and non-participants, and service user group members (see Tables 3, 4 and 5). Some felt that it was too soon and that it would have been preferable to wait until potential participants felt more ready to engage. However, others were concerned that there was only a brief window of opportunity for recruitment following the self-harm presentation, before loss to follow-up. Given that the risk of repeat self-harm and suicide is increased in the period immediately following a self-harm episode, there is a tension between offering help at an early stage or waiting until the person feels more ready to engage. This is likely to vary considerably according to individual preferences and circumstances.

The study age-range (16–30 years) was selected for several reasons. First, up to 60% of self-harm presentations occur in this age range [40]. Second, to determine if the results could be generalised to both adolescents and young adults. Third, to reflect an increasing recent shift in service developments in the UK towards combined mental health services for young people and young adults to overcome barriers to transitions between traditional child and adult services. Fourth and most importantly, this age range is familiar with technology and utilise internet based video-calling (on mobile phones or computer). However, although digital and mobile phone interventions show promise in terms of recruitment of people with depression and anxiety who might not otherwise receive psychological interventions, there were additional barriers for young people with severe depression and recent self-harm. Participants did not necessarily have or could afford consistent mobile phone or internet access. Some reported concerns about data privacy if living with people who were over-controlling or abusive. Poor connectivity of the internet posed an additional barrier in emotionally distressed or poorly motivated participants.

Previous successful RCTs have recruited by simplifying the recruitment process for front-line self-harm assessment staff by using a Zelen design whereby participants are consented only to one form of treatment and data follow-up [41] or participants are recruited independently at follow-up [11]. The former approach is controversial and can lead to imbalances in recruitment if one therapeutic approach in a Zelen design is more popular than the other. The latter study also struggled to recruit participants although it achieved its aims for feasibility.

Alternative study designs might have involved clinicians identifying potential participants on the basis of recent self-harm and the research team assessing for depression and its severity at a later stage or possibly embedding a team of full-time researchers (covering 7 days per week) in each self-harm service to enhance recruitment but the latter would be prohibitively expensive. However, the e-DASH study was commissioned to determine the acceptability and feasibility of an RCT of the effectiveness of an intervention that could be delivered in that same format within NHS services. If the intervention had been found to be effective in real-world clinical settings, it could then potentially have been implemented into routine clinical practice i.e. requiring assessing clinicians to distinguish depression severity without the input of a researcher.

### Implications

Further research is now needed on the validity and clinical utility of routine biopsychosocial assessment soon after a self-harm episode or suicide attempt. It is unclear whether psychosocial assessment, admission to hospital or any other intervention reduces repeat self-harm or suicide rates after self-harm [42]. Our data suggest that people who have self-harmed and have severe or pervasive depression symptoms which precede the crisis leading to the self-harm presentation require further structured psychosocial assessment when they are not emotionally distressed or intoxicated, and this may require admission overnight to facilitate this [1]. Only two of the nine participating services reported using standardised assessments of depression, anxiety, hopelessness and suicide risk. Although previous research with people who have self-harmed indicates that many would like further contact in the period immediately after self-harm [38], only a quarter of adolescents can be reached by health services after self-harm [43]. Only with a considerable amount of effort could we reach 50% of referred participants.

Future research on PSCBT for people with depression and self-harm should consider offering a choice of face-to-face, mobile phone or internet-delivered treatment at a time when participants feel most ready to engage. Such participants might be recruited in the community through primary care and third sector organisations.

## Conclusions

In conclusion, recruitment to RCTs of remotely delivered PSCBT for young people with depression and repeat self-harm is not feasible through recent presentation to clinicians in self-harm services. Offering remotely delivered PSCBT did not enhance the uptake of this intervention in participants aged 16–30 years with depression who had recently presented to medical services following self-harm. Such participants may be recruited more readily in the community once the immediate emotional crisis has passed and the participant is less severely depressed.

## Additional files

Additional file 1: Study Flowchart (DOCX 57 kb)

Additional file 2: Identification of barriers to recruitment and mitigating action (DOCX 17 kb)

Additional file 3: Content of one hour pre-therapy session before starting the problem solving cognitive behaviour therapy (DOCX 16 kb)

Additional file 4: Content and structure of problem solving cognitive behaviour therapy (DOCX 16 kb)

Additional file 5: Efforts made to recruit and retain individuals to the trial by research team (DOCX 18 kb)

Additional file 6: Barriers to recruitment among non-participants, from field notes recorded by research team (DOCX 16 kb)

Additional file 7: Baseline and outcome scores of participants in e-DASH RCT (DOCX 25 kb)

Additional file 8: Attempts to engage participants in the PSCBT intervention (DOCX 18 kb)

Additional file 9: Barriers to mobile phone or video internet delivered problem solving therapy (DOCX 17 kb)

## Abbreviations

BDI-II: Beck Depression Inventory-II
CBT: Cognitive Behaviour Therapy
CSSRS: Columbia Suicide Severity Rating Scale
CTU: Clinical Trials Unit
e-DASH: Electronic - Depression and Self-Harm
GAD-7: Generalised Anxiety Disorder assessment
NHS: National Health Service
NICE: National Institute for Health and Clinical Excellence
PHQ-9: Personal Health Questionnaire
PSCBT: Problem-solving Cognitive Behaviour Therapy
PST: Problem-solving Therapy
RCT: Randomised Controlled Trial
SCID: Structured Clinical Interview for DSM-IV Axis 1 Disorders
TAU: Treatment as usual
UPPS: Urgency Perseverance Premeditation Sensation-seeking
WSAS: Work and Social Adjustment Scale
WTE: Whole Time Equivalent
