# Supplementary material for: Feasibility of a randomised controlled trial of remotely delivered problem-solving cognitive behaviour therapy versus usual care for young people with depression and repeat self-harm: lessons learnt (e-DASH)
Source: BMC Psychiatry. 2019 Jan 24;19:42. doi: 10.1186/s12888-018-2005-3 (PMC6346566; doi:10.1186/s12888-018-2005-3)
Supplement: Supplementary file 1 — Study Flowchart (DOCX 57 kb) [file 12888_2018_2005_MOESM1_ESM.docx]

**Study Flowchart**

Declines to participate in the study = Qualitative interview

Declines to participate in the study = Qualitative interview

Referral into the study by CAMHS / AMHS team who check eligibility criteria

Participants who decline at 3 and 6 months follow-up assessment offered a qualitative interview to find out reasons for drop-out

Researcher completes baseline measures

Analysis of barriers and drivers to the implementation of care carried out alongside data collection

Qualitative interviews with health professionals, managers of services & study participants

Follow-up assessment interviews face to face / video calling / telephone at 3 and 6 months

Allocated to treatment as usual (TAU)

10-12 sessions over 12 weeks with a CBT Therapist via video calling / telephone

Allocated to problem solving cognitive behaviour therapy

Randomisation + Letter sent to inform participant of group allocation

Researcher meets with participant, completes consent form and initial screening = BDI-2 score ≥ 17

Declines to participate in the study = Qualitative interview
