# Supplementary material for: Feasibility of a randomised controlled trial of remotely delivered problem-solving cognitive behaviour therapy versus usual care for young people with depression and repeat self-harm: lessons learnt (e-DASH)
Source: BMC Psychiatry. 2019 Jan 24;19:42. doi: 10.1186/s12888-018-2005-3 (PMC6346566; doi:10.1186/s12888-018-2005-3)
Supplement: Supplementary file 2 — Identification of barriers to recruitment and mitigating action (DOCX 17 kb) [file 12888_2018_2005_MOESM2_ESM.docx]

**Table S2- Identification of barriers to recruitment and mitigating action**

| **Issue/area of concern** | **Barriers** | **Mitigating Action** |
| --- | --- | --- |
| Inclusion Criteria | Concerns were raised by clinicians regarding difficulties in identifying individuals who meet the inclusion criteria of having 2 self-harm episodes in past 12 months. The criteria were considered restrictive and potentially contributing to low recruitment figures. | An amendment was made to extend second self-harm episode to be evident in person’s lifetime. |
|  | Some clinicians were confused about the aspect of the criteria that required the most recent self-harm episode to have occurred in the past 96 hours | An amendment was made to remove the 96 hour requirement, resulting in 4/22 (18%) participants who were referred outside the 96 hour window. |
| Example Participant | Clinicians raised concerns about who would meet the criteria. A suggestion that some example scenarios or vignettes of typical patients would be useful for clinicians to identify people suitable for the study. | Scenario/vignettes developed and circulated to teams. |
| Keeping the study in clinicians’ minds | Clinicians suggested that having an ‘e-DASH’ poster to display in waiting areas/rooms would be very helpful in keeping the study in clinicians’ minds. | Individual posters (site specific) were developed which also included vignette examples (specific for adult and children sites) and were circulated to teams. |
|  | It was felt that embedding a researcher on site at one of the large sites would raise the profile of the study amongst clinicians and allow any queries to be quickly resolved. | A researcher was based in one of the larger adult teams one day a week to act as a point of contact for referring clinicians. |
| Recruitment from acute settings | Concerns were raised that the study may be too much for some participants to take on so soon after an episode of self-harm. | The identification and referral of potential participants through a third sector organisation (who provide support for those actively help-seeking) was put into place which saw recruitment numbers pick up. |
