# Supplementary material for: Feasibility of a randomised controlled trial of remotely delivered problem-solving cognitive behaviour therapy versus usual care for young people with depression and repeat self-harm: lessons learnt (e-DASH)
Source: BMC Psychiatry. 2019 Jan 24;19:42. doi: 10.1186/s12888-018-2005-3 (PMC6346566; doi:10.1186/s12888-018-2005-3)
Supplement: Supplementary file 3 — Content of one hour pre-therapy session before starting the problem solving cognitive behaviour therapy (DOCX 16 kb) [file 12888_2018_2005_MOESM3_ESM.docx]

**Content of one hour pre-therapy session before starting the problem solving cognitive behaviour therapy**

- The boundaries of confidentiality were discussed with the participant. A prerequisite of inclusion in the study was that each participant had to provide the name and telephone numbers of up to three people the study team could contact if deemed necessary in terms of risk
- An outline of the proposed intervention was shared with the participant
- A mood rating was completed using the 9-item Personal Health Questionnaire (PHQ-9, Kroenke et al, 2001)
- A preliminary clinical risk assessment was conducted
- A safety plan was developed, the specifics of which varied according to the level of risk elicited in the risk assessment
- The participant was asked to choose their preferred mode of delivery
- Any questions the participant had regarding the study were answered
