# Supplementary material for: Feasibility of a randomised controlled trial of remotely delivered problem-solving cognitive behaviour therapy versus usual care for young people with depression and repeat self-harm: lessons learnt (e-DASH)
Source: BMC Psychiatry. 2019 Jan 24;19:42. doi: 10.1186/s12888-018-2005-3 (PMC6346566; doi:10.1186/s12888-018-2005-3)
Supplement: Supplementary file 4 — Content and structure of problem solving cognitive behaviour therapy (DOCX 16 kb) [file 12888_2018_2005_MOESM4_ESM.docx]

**Content and structure of problem solving cognitive behaviour therapy**

The content and structure of the protocol for the intervention followed the Brown and Beck (2005) protocol and consisted of 10-12 sessions, each of 60 minutes duration. The frequency of sessions was weekly or bi-weekly but could be increased in frequency according to clinical need. This included the following:

- Engagement in the therapy process and sharing a treatment rationale
- Identifying past and current triggers for self-harm episodes and suicide attempts
- Making a cognitive formulation of thoughts, images and core beliefs activated prior to self-harm episode or suicide attempt
- Behavioural and cognitive interventions to address these thoughts, images and beliefs
- Problem-solving strategies to deal with stressors
- Specific vulnerability factors were targeted specifically hopelessness, impulsivity, poor problem–solving and social isolation.
- Safety plans were developed as outlined in the protocol developed by Brown et al (2002).

The session structure followed that of standard CBT clinical practice (Kinsella and Garland 2008) and included an active, focused problem-solving approach to tackling difficulties. The therapy style used collaborative empiricism, Socratic questioning and guided discovery (Beck et al 1979). Agenda setting was used to structure the content of each session and homework was agreed and reviewed each session.
