# Supplementary material for: Feasibility of a randomised controlled trial of remotely delivered problem-solving cognitive behaviour therapy versus usual care for young people with depression and repeat self-harm: lessons learnt (e-DASH)
Source: BMC Psychiatry. 2019 Jan 24;19:42. doi: 10.1186/s12888-018-2005-3 (PMC6346566; doi:10.1186/s12888-018-2005-3)
Supplement: Supplementary file 5 — Efforts made to recruit and retain individuals to the trial by research team (DOCX 18 kb) [file 12888_2018_2005_MOESM5_ESM.docx]

**Table S1 - Efforts made to recruit and retain individuals to the trial by research team**

| **Time Point** | **Eligible Participants** | **No. of phone calls made** | **Mean no. of phone calls made pp** | **No. of text messages sent** | **Mean no. of text messages sent pp** | **No. of answer phone messages left** | **Mean no. of answer phone messages left pp** | **No. of times NCP contacted** | **No of cancelled/no show appointments** | **No of letters sent** |
| --- | --- | --- | --- | --- | --- | --- | --- | --- | --- | --- |
| **Baseline** | 43 | 109 | 2.66 | 31 | 0.76 | 39 | 0.95 | 2 | 19 | 0 |
| **3 month** | 22 | 42 | 1.91 | 18 | 0.82 | 16 | 0.73 | 1 | 2 | 0 |
| **6 month** | 21 | 29 | 1.38 | 12 | 0.57 | 7 | 0.33 | 1 | 5 | 0 |
| **9 month** | 10 | 19 | 1.90 | 10 | 1.00 | 8 | 0.80 | 0 | 2 | 0 |
| **12 month** | 9 | 15 | 1.67 | 4 | 0.44 | 6 | 0.67 | 0 | 0 | 0 |
| **Post Interview** | 18 | 22 | 1.11 | 20 | 1.00 | 4 | 0.22 | 0 | 1 | 6 |
| **Total** |  | **236** |  | **95** |  | **80** |  | **4** | **30** | **6** |

pp = per person

NCP = nominated contact persons (three named people whom participants gave the study team permission to contact in the face of escalating risk e.g. unable to contact and frequent or intrusive suicide ideation mentioned at previous contact)
