# Supplementary material for: Feasibility of a randomised controlled trial of remotely delivered problem-solving cognitive behaviour therapy versus usual care for young people with depression and repeat self-harm: lessons learnt (e-DASH)
Source: BMC Psychiatry. 2019 Jan 24;19:42. doi: 10.1186/s12888-018-2005-3 (PMC6346566; doi:10.1186/s12888-018-2005-3)
Supplement: Supplementary file 6 — Barriers to recruitment among non-participants, from field notes recorded by research team (DOCX 16 kb) [file 12888_2018_2005_MOESM6_ESM.docx]

**Barriers to recruitment among non-participants, from field notes recorded by research team**

• Criteria Issues: One referral did not fall within the age inclusion criteria i.e. referred in error.

• Personal Reasons/Wrong Time: Two individuals did not wish to be considered for the study after cancelling their baseline visits (one due to a death in the family and the other repeatedly cancelled (or forgot), then withdrew completely, due to an unspecified issue in their personal life). One individual cancelled on the day of their baseline visit citing personal reasons and said they would get in touch if they wanted to be involved again. Another individual (who had given their parent as the study contact) told us to call back at a later date.

• Contact Issues: One individual (who gave their parent’s mobile number as the contact method) said we had the wrong number when we called. Two individuals’ contact numbers were not receiving calls or were turned off (one of whom cancelled their initial baseline appointment as they were “not doing well” at that present time). Another individual, after arranging baseline, was no longer living at the address that was given and the contact number was no longer in operation.
