# Supplementary material for: Feasibility of a randomised controlled trial of remotely delivered problem-solving cognitive behaviour therapy versus usual care for young people with depression and repeat self-harm: lessons learnt (e-DASH)
Source: BMC Psychiatry. 2019 Jan 24;19:42. doi: 10.1186/s12888-018-2005-3 (PMC6346566; doi:10.1186/s12888-018-2005-3)
Supplement: Supplementary file 7 — Baseline and outcome scores of participants in e-DASH RCT (DOCX 25 kb) [file 12888_2018_2005_MOESM7_ESM.docx]

**Table S3 - Baseline and outcome scores of participants in e-DASH RCT**

| **Outcome Measure** | | **BDI-II** | | **PHQ-9** | | **CSSRS**  **(ideation)** | | **Beck Hopelessness**  **Scale** | | **GAD-7** | | **WSAS** | |
| --- | --- | --- | --- | --- | --- | --- | --- | --- | --- | --- | --- | --- | --- |
| *Study arm* | | TAU | PSCBT + TAU | TAU | PSCBT + TAU | TAU | PSCBT + TAU | TAU | PSCBT + TAU | TAU | PSCBT + TAU | TAU | PSCBT + TAU |
| **Baseline** | Mean  sd  Median  Min  Max  n (%) | 37.2  11.0  32  26  56  11 (100) | 38.9  13.1  34  18  56  11 (100) | 19.3  4.9  22  11  26  11 (100) | 19.9  5.3  20  11  27  11 (100) | 3.3  1.9  11 (100) | 3.2  1.6  11 (100) | 13.8  5.8  15  6  20  11 (100) | 13.7  5.3  13  3  20  11 (100) | 14.7  3.8  15  9  21  11 (100) | 14.0  5.6  14  7  20  11 (100) | 20.1  10.5  21  4  39  11 (100) | 24.9  8.9  24  8  37  11 (100) |
| **3 month** | Mean  sd  Median  Min  Max  n (%) | 30.0  21.1  35  0  56  5 (45.5) | 23.5  13.2  26.5  2  37  6 (54.5) | 16.8  11.1  20  1  27  5 (45.5) | 13.0  7.7  16.5  1  24  6 (54.5) | 3.6  2.2  5 (45.5) | 3.6  1.9  6 (54.5) | 12.4  6.7  13  2  20  5 (45.5) | 11.4  7.2  13  1  20  6 (54.5) | 8.6  7.9  8  0  21  5 (45.5) | 12.0  8.8  14.5  0  19  6 (54.5) | 20.8  14.9  21  0  40  5 (45.5) | 16.7  10.2  18  0  28  6 (54.5) |
| **6 month** | Mean  sd  Median  Min  Max  n (%) | 27.7  22.3  19  11  53  3 (27.3) | 18.7  14.2  19  0  44  7 (63.6) | 15.3  9.5  12  8  26  3 (27.3) | 11.9  8.0  12  0  22  7 (63.6) | 3.9  1.0  3 (27.3) | 3.0  1.3  7 (63.6) | 12.1  6.9  9.4  7  20  3 (27.3) | 9.5  6.0  9.5  1  20  7 (63.6) | 12.0  7.9  9  6  21  3 (27.3) | 9.9  7.5  7  1  21  7 (63.6) | 18.7  17.6  9  8  39  3 (27.3) | 16.3  15.8  21  0  40  7 (63.6) |
| **9 month** | Mean  sd  Median  Min  Max  n (%) | -  -  0 (0) | 27.0  19.9  16  15  50  3 (27.3) | -  -  0 (0) | 14.0  7.0  14  7  21  3 (27.3) | -  -  0 (0) | 1.7  1.2  3 (27.3) | -  -  0 (0) | 10.0  8.9  7  3  20  3 (27.3) | -  -  0 (0) | 15.2  3.6  17  11  18  3 (27.3) | -  -  0 (0) | 24.7  11.0  30  12  32  3 (27.3) |
| **12 month** | Mean  sd  Median  Min  Max  n (%) | -  -  0 (0) | 15.7  10.6  14  6  27  3 (27.3) | -  -  0 (0) | 11.0  6.6  10  5  18  3 (27.3) | -  -  0 (0) | 1.0  1.7  3 (27.3) | -  -  0 (0) | 8.0  6.1  11  1  12  3 (27.3) | -  -  0 (0) | 4.0  1.4  4  3  5  3 (27.3) | -  -  0 (0) | 3.3  4.2  2  0  8  3 (27.3) |

BDI-II = Beck depression Inventory version 2; PHQ-9 = Personal Health Questionnaire, 9-items;

CSSRS = Columbia Suicide Severity Rating Scale; GAD-7 = Generalised Anxiety Disorder rating scale, 7 item

WSAS = Work and Social Adjustment Scale

TAU = Treatment as usual; PSCBT + TAU = Problem solving CBT and treatment as usual.
