# Supplementary material for: Feasibility of a randomised controlled trial of remotely delivered problem-solving cognitive behaviour therapy versus usual care for young people with depression and repeat self-harm: lessons learnt (e-DASH)
Source: BMC Psychiatry. 2019 Jan 24;19:42. doi: 10.1186/s12888-018-2005-3 (PMC6346566; doi:10.1186/s12888-018-2005-3)
Supplement: Supplementary file 8 — Attempts to engage participants in the PSCBT intervention (DOCX 18 kb) [file 12888_2018_2005_MOESM8_ESM.docx]

**Table S4 - Attempts to engage participants in the PSCBT intervention**

| Participant |  |  | Number of phone calls | Number non-response to phone calls | Number of voice messages left | Number of text messages sent | Number of non-responses to text messages | Number of emails sent |
| --- | --- | --- | --- | --- | --- | --- | --- | --- |
| 1 |  |  | 33 | 10 | 0 | 4 | 4 | 3 |
| 2 |  |  | 28 | 20 | 20 | 4 | 4 | 0 |
| 3 |  |  | 30 | 17 | 15 | 4 | 4 | *n/a |
| 4 |  |  | 52 | 46 | 30 | 9 | 6 | *n/a |
| 5 |  |  | 32 | 19 | 19 | 6 | 0 | 4 |
| 6 |  |  | 12 | 7 | 0 | 3 | 0 | 0 |
| 7 |  |  | 40 | 30 | 30 | 5 | 3 | 1 |
| 8 |  |  | 32 | 19 | 19 | 6 | 0 | 5 |
| 9 |  |  | 15 | 6 | *n/a | *n/a | 0 | 13 |
| 10 |  |  | 25 | 10 | 5 | 0 | 0 | 8 |
| Total |  |  | **299** | **184** | **138** | **41** | **21** | **34** |

* participant opted out of this method of contact

NB: There is no column indicating number of non-responses to emails because all emails received a response
