# Supplementary material for: Feasibility of a randomised controlled trial of remotely delivered problem-solving cognitive behaviour therapy versus usual care for young people with depression and repeat self-harm: lessons learnt (e-DASH)
Source: BMC Psychiatry. 2019 Jan 24;19:42. doi: 10.1186/s12888-018-2005-3 (PMC6346566; doi:10.1186/s12888-018-2005-3)
Supplement: Supplementary file 9 — Barriers to mobile phone or video internet delivered problem solving therapy (DOCX 17 kb) [file 12888_2018_2005_MOESM9_ESM.docx]

**Barriers to mobile phone or video internet delivered problem solving therapy**

| • Severity of depression e.g. for participant 5, this interfered with ability to utilise intervention.  • Trying to engage with the intervention at the time of crisis which participants indicated added additional burden to already existing stressors e.g. participant 3 who dropped out after 3 telephone sessions.  • Social isolation e.g. participant 3 had been a Looked-After child and since leaving care at age 18 had no family or social network.  • Shared phone usage e.g. participant 4 only had access to a mobile phone on alternate weeks as they shared this with their partner.  • Restricted finances to fund mobile phone e.g. participant 1 took longer than expected to complete the treatment as there were times that they could not use their phone because of finances.  • Shared computer on which parents could access participant’s emails e.g. participant 7.  • Other health professional advised a participant that CBT was not an appropriate intervention for them e.g. participant 7.  • First choice of method of remote delivery problematic e.g. participants 5, 6, 8, 9, 10 Broadband speed too slow for WebeX to function as intended. |
| --- |

Note: Barriers were multiple for three participants; no barriers in only one participant; one participant withdrawn for safety reasons before starting PSCBT.
